# Comparison of Midwife-Led and Obstetrician-Led Care Models: Associations with Maternal Bonding and Breastfeeding Self-Efficacy

**DOI:** 10.3390/healthcare14203487

**Published:** 2026-10-17

**Authors:** Milda Naginevičiūtė, Eglė Bartusevičienė, Aurelija Blaževičienė

**Affiliations:** 1 Department of Nursing, Faculty of Nursing, Lithuanian University of Health Sciences, LT 44307 Kaunas, Lithuania; aurelija.blazeviciene@lsmu.lt; 2 Department of Obstetrics and Gynecology, Faculty of Medicine, Lithuanian University of Health Sciences, LT 44307 Kaunas, Lithuania; egle.bartuseviciene@lsmu.lt

**Keywords:** midwife-led care, mother–infant bonding, breastfeeding self-efficacy, patient-reported outcomes

## Abstract

**Background/Objectives:** The postpartum period is important for mother–infant bonding and breastfeeding self-efficacy. This study compared these outcomes between women receiving midwife-led and obstetrician-led intrapartum care. **Methods:** A prospective longitudinal observational study was conducted in two Lithuanian hospitals between September 2022 and July 2024. Women with low-risk births (n = 349) were assessed at 1–3 days and 6–8 weeks postpartum using the MIBS and BSES-SF. **Results:** In unadjusted comparisons, total MIBS scores showed a more stable pattern in the MW-led group (between-group difference in change, *p* = 0.004), and total BSES-SF scores increased within the MW-led group (*p* < 0.001). In adjusted analyses, the care setting was not independently associated with change in total MIBS or total BSES-SF scores. At 6–8 weeks postpartum, care setting (*p* = 0.041) and parity (*p* = 0.017) were associated with total BSES-SF scores. **Conclusions:** Selected unadjusted outcomes showed more favourable patterns in the MW-led setting, but adjusted analyses did not support an independent association between care setting and change in either principal outcome. Since each model was represented by a different hospital, observed associations cannot be attributed specifically to the professional care model.

## 1. Introduction

The transition to motherhood is a complex biological, psychological, and social process [1]. Its success depends not only on the woman’s personal experience but also on the institutional environment in which it occurs [2,3]. One of the most important phases of this transition is the postpartum period, during which a woman adjusts to her new role, establishes an emotional bond with her baby, gains confidence in caring for the newborn, and shapes her maternal identity [4,5].

Research suggests that these processes are associated with the nature of care provided during childbirth, the professional background of the healthcare provider, the quality of communication, and the extent to which the woman is involved in decision-making [5,6,7,8,9]. Although in many countries maternity care is still primarily led by obstetricians, increasing attention is being paid to whether this model truly addresses the holistic needs of women. In contrast, midwife-led models—especially those based on continuity of care—are characterized by a stronger focus on the woman, closer emotional connection between the midwife and the woman, and greater empowerment [10]. The World Health Organization has long recommended midwife-led care for healthy pregnancies, highlighting its benefits for both maternal and newborn health, psychological well-being, and care continuity [10].

Studies have shown that midwife-led care is associated with higher maternal satisfaction, fewer medical interventions, improved emotional outcomes after childbirth, and better breastfeeding results [10,11]. However, the implementation of these models varies depending on how maternity services are structured and on the level of professional autonomy granted to midwives [6]. In countries where midwives practice independently, such as the United Kingdom or the Netherlands, these models are well established [11,12]. In contrast, in Lithuania, midwives’ autonomy remains limited, and their potential in maternity care is underutilized [13]. Although midwives are legally authorized to independently manage low-risk births, including conducting vaginal deliveries [13], in practice obstetricians frequently continue to attend such deliveries, and midwives’ autonomous role is not fully recognized. As a result, different models of intrapartum care coexist, with some settings applying a midwife-led approach and others maintaining an obstetrician-led model.

In order to develop woman-centred, evidence-based maternity services, it is necessary to move beyond structural system analysis and include empirical assessments of women’s experiences [14]. Increasingly, researchers emphasize that clinical indicators such as complication rates or perinatal survival are not sufficient to assess the quality of care from the patient’s perspective [15,16,17]. It is equally important to understand how women experience childbirth, how the mother–infant bond develops, and how confident they feel in breastfeeding and caring for their newborn [18,19]. To capture these dimensions, patient-reported outcome measures (PROMs) are increasingly used [18,19]. These tools allow for the assessment of a woman’s emotional adjustment, self-confidence, and other subjective yet clinically important aspects of the postpartum period [19,20]. PROMs provide deeper insights into women’s needs and expectations, help evaluate care models and service quality, and can inform policy development [20,21]. Importantly, they promote a feedback loop between the woman’s experience and service provision, supporting care that is not only safe but also emotionally supportive and empowering [22].

Despite the growing attention to maternal experiences in the postpartum period, there is a lack of empirical studies comparing associations between different care settings, emotional adaptation, and self-efficacy [20,22]. This gap is particularly evident in settings where midwifery autonomy is still limited [10,12,20].

This study aims to compare postpartum outcomes between women who received midwife-led and obstetrician-led care, with a focus on mother–infant bonding and breastfeeding self-efficacy.

## 2. Materials and Methods

### 2.1. Study Design and Participants

This prospective longitudinal observational study was conducted between September 2022 and July 2024. The study targeted postpartum women aged 18 years or older who had undergone a low-risk delivery and were fluent in Lithuanian. Women whose deliveries initially met low-risk criteria but developed complications during labor or birth were excluded. Of the 349 participants, 169 (48.4%) gave birth in a midwife-led care (MW-led) setting, and 180 (51.6%) in an obstetrician-led care (OB-led) setting. The sample size was estimated using the standard formula for comparing two independent means, considering the annual number of approximately 300 low-risk births in each hospital. The required sample was calculated to be 170 participants per group, and our achieved sample (MW-led n = 169; OB-led n = 180) met this requirement, ensuring sufficient statistical power to detect clinically meaningful differences.

Data were collected in two Lithuanian hospitals representing distinct models of intrapartum care: a university hospital providing midwife-led care for low-risk deliveries, and a regional hospital where deliveries are managed by obstetrician-gynecologists. In the midwife-led setting, qualified midwives independently oversee intrapartum care for women classified as low-risk. In the obstetrician-led model, care is provided by obstetrician-gynecologists. Although both hospitals cared for women with low-risk deliveries, their models of care reflected different professional roles. In the university hospital, midwives provided intrapartum care independently for women with low-risk pregnancies. In contrast, in the regional hospital, obstetricians remained the primary care providers for all deliveries, and midwives worked mainly in supportive roles. Both hospitals were accredited as Baby-Friendly Hospitals at the time of the study. In line with the Baby-Friendly Hospital Initiative, lactation consultants were available in both settings and routinely visited all mothers after childbirth to provide breastfeeding counseling and support. Thus, structured breastfeeding support was consistently ensured across the two study sites. Because the care model was fully aligned with hospital, associations with care setting cannot be disentangled from other institutional characteristics in this study.

Participants were identified and invited during their hospital stay shortly after childbirth. Data were collected at two time points to assess maternal experiences during the postpartum period: T1 (within 1–3 days postpartum, using paper-based questionnaires administered in the hospital), T2 (at 6–8 weeks postpartum, via email using an online survey). The same cohort of women was followed across both time points at the same healthcare institutions.

### 2.2. Research Instrument

This study employed two standardized, self-report instruments to assess maternal experiences in the postpartum period: the Mother–Infant Bonding Scale (MIBS) and the Breastfeeding Self-Efficacy Scale—Short Form (BSES-SF). The selected instruments were drawn from the Pregnancy and Childbirth outcome set developed by the International Consortium for Health Outcomes Measurement (ICHOM), which recommends the use of validated patient-reported outcome measures to assess bonding, and self-efficacy in the postnatal period [23].

The MIBS is an 8-item scale used to identify potential bonding difficulties between mother and infant. Each item is scored on a 4-point Likert scale (0–3), with total scores ranging from 0 to 24; higher scores indicate more emotional difficulties in bonding [24].

The BSES-SF consists of 14 items that assess maternal self-efficacy related to breastfeeding. Items are rated on a 5-point Likert scale, with a total score range of 14 to 70; higher scores reflect greater confidence in breastfeeding ability [25].

Both questionnaires were translated from English into Lithuanian following the ISPOR Principles of Good Practice for the translation and cultural adaptation of patient-reported outcome measures. The process included forward translation, back-translation, expert review, and pilot testing among postpartum women to ensure clarity, conceptual equivalence, and cultural appropriateness. In the present sample, internal consistency was high for both instruments. Cronbach’s alpha was 0.857 at 1–3 days and 0.884 at 6–8 weeks postpartum for the MIBS, and 0.927 at 1–3 days and 0.976 at 6–8 weeks postpartum for the BSES-SF.

### 2.3. Statistical Analysis

Data were analyzed using the Statistical Package for Social Sciences (SPSS for Windows 30.0). Descriptive statistics were used to summarize the data. The principal outcomes were the total MIBS and total BSES-SF scores and their changes between T1 and T2. Item-level analyses were considered exploratory and were performed to provide additional detail on specific aspects of mother–infant bonding and breastfeeding self-efficacy. Categorical variables are reported as absolute frequencies (n) and percentages (%); continuous variables are presented as means (M) with standard deviations (SD). The normality of data distribution was assessed using the Kolmogorov–Smirnov test. As several variables did not meet the assumptions of normality, non-parametric tests were applied. Group differences in ordinal variables were assessed using the Mann–Whitney U test for independent samples and the Wilcoxon signed-rank test for paired samples. Change (Δ) scores were calculated for participants with available data at both T1 and T2 (paired complete cases) as the within-participant difference between T2 and T1. Descriptive means at T1 and T2 were calculated using all available observations at each respective time point. Therefore, the mean Δ does not necessarily equal the arithmetic difference between the separately calculated T1 and T2 group means. Missing data were handled using available-case analysis, with no imputation performed. Between-group differences in Δ scores were assessed using the Mann–Whitney U test. For item-level analyses, unadjusted *p*-values are reported. To account for multiple comparisons, statistical significance was evaluated using Bonferroni-adjusted thresholds of *p* < 0.00625 for the eight MIBS items (0.05/8) and *p* < 0.00357 for the 14 BSES-SF items (0.05/14).

For post hoc comparisons within sociodemographic variables, the z-test for proportions with Bonferroni correction was used. Effect sizes were calculated using Cohen’s *d* and interpreted as follows: <0.2—negligible effect, 0.2—small effect, 0.5—medium effect, and 0.8—large effect. To identify predictors of outcomes, multivariable linear regression analyses were performed, and results are presented as unstandardized (B) and standardized (β) coefficients with 95% confidence intervals (CIs). For analyses not subject to correction for multiple comparisons, *p* < 0.05 was considered statistically significant. For item-level MIBS and BSES-SF analyses, the Bonferroni-adjusted significance thresholds specified above were applied. Values of *p* < 0.001 are reported as such rather than as *p* = 0.000.

### 2.4. Ethical Considerations

The study was approved by the Biomedical Research Ethics Committee of the Kaunas Region (approval date: 21 July 2022, No. P1-BE-2-24/2022). Participation in the study was voluntary, and informed consent was obtained from all participants prior to their inclusion. Women who agreed to participate signed an informed consent form before completing the first questionnaire. All data were processed in accordance with ethical principles and data protection regulations to ensure participants’ privacy and confidentiality.

## 3. Results

A total of 349 postpartum women participated at baseline: 169 (48.4%) in the midwife-led (MW-led) setting and 180 (51.6%) in the obstetrician-led (OB-led) setting. Sociodemographic characteristics were broadly similar, except for education (Table 1). The principal outcomes were total MIBS and BSES-SF scores and their T1–T2 changes; item-level comparisons are presented as exploratory findings.

Participant follow-up: paired T1–T2 data were available for 94 of 169 women in the MW-led group (55.6%) and 124 of 180 in the OB-led group (68.9%), for a total of 218 paired observations. Thus, 75 MW-led and 56 OB-led participants did not contribute to paired change-score analyses. The original analysis reported no statistically significant between-group difference in attrition (binomial test, all *p* > 0.05); however, the numerical difference in retention should be considered when interpreting longitudinal comparisons. Descriptive T1 and T2 scores use all observations available at each time point, whereas change scores use paired complete cases.

The primary MIBS analysis concerned the total score and its change over time. Lower MIBS scores indicate fewer mother–infant bonding difficulties; accordingly, increases represent less favourable bonding scores. In unadjusted analyses, total MIBS scores increased in the OB-led group (*p* = 0.001), while the change in the MW-led group was not statistically significant (*p* = 0.058). The unadjusted between-group comparison of total-score change was significant (*p* = 0.004; Cohen’s *d* = 0.25; Table 2). Exploratory item-level analyses, interpreted using a Bonferroni threshold of *p* < 0.00625, identified changes in selected items, including a between-group difference in change for “neutral or feeling nothing” (*p* = 0.001). These exploratory findings do not supersede the adjusted total-score analysis.

To further account for potential confounders, we performed a multivariable linear regression analysis for the change in total MIBS scores between T1 and T2 (Δ MIBS). As shown in Table 3, none of the predictors (model of intrapartum care, maternal age, educational level, marital status, parity, number of people providing support, pre-pregnancy BMI) reached statistical significance.

Total BSES-SF scores and their change between T1 and T2 were the principal breastfeeding self-efficacy outcomes; higher scores indicate greater self-efficacy. In unadjusted analyses, total BSES-SF scores increased within the MW-led group (*p* < 0.001) and the OB-led group (*p* = 0.004), and the between-group difference in change was statistically significant (*p* = 0.017; Table 4). Exploratory item-level analyses used the Bonferroni-adjusted threshold of *p* < 0.00357 and suggested changes in selected breastfeeding-confidence items in both settings. These item-level observations should not be interpreted as evidence of an independent care-model association with the change in total BSES-SF scores.

To further explore predictors of breastfeeding self-efficacy, we conducted a multivariable linear regression for the change in total BSES-SF scores between T1 and T2 (Δ BSES-SF). As shown in Table 5, the number of people providing support (β = 0.16, *p* = 0.018) and pre-pregnancy BMI (β = 0.14, *p* = 0.045) were associated with greater improvements in breastfeeding self-efficacy. No other predictors reached statistical significance.

To further explore predictors of breastfeeding self-efficacy at T2, we performed a multivariable linear regression analysis (Table 6).

At T2, care setting (β = 0.14, *p* = 0.041) and parity (β = 0.17, *p* = 0.017) were associated with total BSES-SF scores in the adjusted model (Table 6). This cross-sectional association at T2 should be distinguished from the non-significant adjusted association of care setting with longitudinal change in total BSES-SF scores (Table 5). Because care setting coincided with hospital, the T2 association cannot be ascribed specifically to the professional model of care.

## 4. Discussion

This study compared mother–infant bonding and breastfeeding self-efficacy in two Lithuanian hospitals with distinct intrapartum care models. The principal findings concern total MIBS and BSES-SF scores and their changes over time. Although selected unadjusted comparisons favoured the MW-led setting, the care setting was not independently associated with change in either total score after adjustment for measured covariates. In a separate adjusted analysis at 6–8 weeks postpartum, care setting and parity were associated with the total BSES-SF score. These findings must be interpreted as associations between hospital-based settings and reported postpartum outcomes, not causal effects of the professional care model. In particular, hospital identity and care model were completely aligned and could not be analysed separately.

For bonding, the unadjusted total MIBS score remained relatively stable in the MW-led group and increased in the OB-led group. Because higher MIBS scores indicate more bonding difficulties, this was a more favourable unadjusted pattern in the MW-led setting. However, the adjusted model for change in the total MIBS score did not identify an independent association with care setting. The isolated MIBS item findings were exploratory and should be interpreted in light of the multiple-comparison correction. The previous literature describes associations between supportive birth experiences and maternal emotional adaptation [26,27,28,29,30], but the present design cannot determine whether differences in bonding trajectories were related to the professional model, institutional practices, or unmeasured participant characteristics.

For breastfeeding self-efficacy, total BSES-SF scores increased in both settings in unadjusted within-group comparisons, with a larger unadjusted change in the MW-led group. Nevertheless, the adjusted change-score analysis did not identify an independent association with care setting. The number of people providing support and pre-pregnancy BMI were associated with change in total BSES-SF scores, while care setting and parity were associated with total BSES-SF scores at T2. Exploratory item analyses suggested improvements in selected practical breastfeeding-confidence domains, including latch and feeding management, but should not be emphasised over the findings for total scores. The existing literature highlights the relevance of supportive, woman-centred maternity care for breastfeeding experiences [31,32,33,34,35]; these data do not identify which aspects of support, if any, explain differences between the two hospitals.

The association between care setting and total BSES-SF at 6–8 weeks is a finding at one follow-up time point, not evidence that a particular model led to greater improvement over time. Psychological distress and postpartum depression were not measured; conclusions about their prevention or reduction are therefore unwarranted. Findings from prior research on care organisation and maternal wellbeing provide context, rather than a causal explanation for these results [10,29,36,37].

Lithuania provides an informative context because professional responsibilities in maternity care vary across institutions. The present results describe postpartum outcomes in two specific hospital settings. They may inform questions for service evaluation, but they do not establish comparative effectiveness of professional care models.

Future studies should include multiple hospitals for each care model, collect detailed information about the care actually received, and account for both institutional and patient-level differences. This would help clarify whether the associations observed here are reproducible beyond these two settings.

### Strengths and Limitations

The study followed women at two postpartum time points and used established patient-reported instruments, the MIBS and BSES-SF, to assess clinically relevant experiences beyond routine biomedical outcomes. It also contributes evidence from a setting in which different intrapartum care arrangements coexist.

Several limitations materially constrain interpretation. First, the most important limitation is complete confounding between hospital and professional care models: only one hospital represented each model. Institutional culture, staffing, referral pathways, postnatal routines, and unmeasured patient characteristics may therefore explain some or all observed differences, and regression adjustment cannot isolate a care-model association from a hospital association. Second, the longitudinal analysis relied on paired complete cases and change-score regressions rather than mixed-effects or generalized estimating equation (GEE) models, which would have been more appropriate to account for repeated observations and patterns of incomplete follow-up. Only 94/169 women in the MW-led group and 124/180 in the OB-led group contributed to paired analyses; the difference in retention and possible selection bias warrant caution, regardless of the reported non-significant attrition test. Third, adjustment covered a limited set of measured covariates; prior breastfeeding experience, mental health, detailed infant health, and wider contextual factors were unavailable. Exploratory item-level testing also increases the risk of chance findings despite Bonferroni thresholds. Taken together, these limitations preclude causal interpretation and restrict generalisability beyond the participating hospitals.

## 5. Conclusions

In this two-hospital observational study, the MW-led setting showed more favourable unadjusted patterns in selected measures of mother–infant bonding and breastfeeding self-efficacy. Lower total MIBS scores indicate fewer bonding difficulties. However, care setting was not independently associated with change in total MIBS or total BSES-SF scores after adjustment. Care setting and parity were associated with total BSES-SF scores at 6–8 weeks, whereas social support and pre-pregnancy BMI were associated with change in total BSES-SF scores. Because each care model was represented by a different hospital and repeated-measures modelling was limited, these associations cannot establish effects attributable to the professional model of care. Multicentre longitudinal studies are needed.

## Figures and Tables

**Table 1:** Characteristics of the participants.

Characteristics	MW-Led (n = 169)	OB-Led (n = 180)	Overall
			(n = 349)
Maternal age (mean (SD))	30.57 (5.2)	30.64 (4.2)	30.61 (4.7)
Educational level			
Primary/Secondary/Vocational	57 (33.7)	36 (20.0)	93 (26.6)
Higher non-university	81 (47.9)	108 (60.0)	189 (54.2)
Higher university	31 (18.3)	36 (20.0)	67 (19.2)
Marital status			
Married	131 (77.5)	148 (82.2)	279 (79.9)
Cohabiting	35 (20.7)	30 (16.7)	65 (18.6)
Single/Divorced	3 (1.8)	2 (1.1)	5 (1.4)
Parity			
Nulliparous	95 (56.2)	104 (57.8)	199 (57.0)
Multiparous	74 (43.8)	76 (42.2)	150 (43.0)
Number of people providing support			
1	4 (2.4)	10 (5.6)	14 (4.0)
2–5	114 (67.5)	124 (68.9)	238 (68.2)
6–10	51 (30.2)	46 (25.6)	97 (27.8)
Pre-pregnancy body mass index (kg/m^2^)			
<18.5	19 (11.2)	8 (4.4)	27 (7.7)
18.5–24.9	104 (61.5)	109 (60.6)	213 (61.0)
25.0–29.9	30 (17.8)	39 (21.7)	69 (19.8)
≥30	16 (9.5)	24 (13.3)	40 (11.5)

Values are numbers (percentages) unless otherwise stated.

**Table 2:** Changes in mother–infant bonding between midwife-led and obstetrician-led care groups.

MIBS Statement		T1 (Mean ± SD)	T2 (Mean ± SD)	*p* (T1 vs. T2) *	Δ (Mean ± SD)	Cohen’s *d* (Δ)	*p* (Δ Comparison) **
Loving	OB-led	0.07 (0.3)	0.09 (0.4)	0.109	0.01 (0.5)	0.13	0.043
	MW-led	0.08 (0.3)	0.04 (0.2)	0.006	−0.05 (0.3)		
Full of resentment	OB-led	0.06 (0.2)	0.21 (0.6)	0.002	0.15 (0.6)	0.14	0.030
	MW-led	0.09 (0.3)	0.15 (0.6)	0.061	0.05 (0.7)		
Neutral or feeling nothing	OB-led	0.07 (0.3)	0.15 (0.4)	0.010	0.07 (0.6)	0.36	0.001
	MW-led	0.11 (0.5)	0.06 (0.2)	0.006	−0.12 (0.6)		
Joyful	OB-led	0.25 (0.5)	0.42 (0.6)	0.002	0.15 (0.7)	0.16	0.040
	MW-led	0.32 (0.6)	0.38 (0.6)	0.081	0.03 (0.8)		
Negative	OB-led	0.05 (0.2)	0.24 (0.7)	<0.001	0.18 (0.6)	0.17	0.012
	MW-led	0.12 (0.4)	0.16 (0.4)	0.108	0.01 (0.7)		
Protective	OB-led	0.26 (0.5)	0.25 (0.5)	0.062	−0.05 (0.7)	0.04	0.049
	MW-led	0.25 (0.5)	0.18 (0.5)	0.024	−0.07 (0.7)		
Disappointed	OB-led	0.07 (0.3)	0.14 (0.3)	0.025	0.05 (0.4)	−0.19	0.021
	MW-led	0.09 (0.3)	0.22 (0.4)	<0.001	0.13 (0.3)		
Aggressive	OB-led	0.01 (0.1)	0.05 (0.2)	0.002	0.05 (0.2)	0.13	0.044
	MW-led	0.04 (0.2)	0.06 (0.2)	0.040	0.02 (0.2)		
Total	OB-led	0.83 (1.3)	1.55 (2.3)	0.001	0.60 (2.3)	0.25	0.004
	MW-led	1.11 (1.8)	1.27 (1.8)	0.058	−0.01 (2.4)		

* Wilcoxon signed-rank test; ** Mann–Whitney U test. Unadjusted *p*-values are reported. For item-level comparisons, statistical significance was evaluated using a Bonferroni-adjusted threshold of *p* < 0.00625 (0.05/8). Paired T1–T2 analyses included 94 women in the MW-led group and 124 women in the OB-led group. T1 and T2 descriptive means are based on all available observations at each time point, whereas Δ scores and paired within-group comparisons are based only on participants with data available at both T1 and T2.

**Table 3:** Multivariable linear regression for changes (Δ T2–T1) in total MIBS scores.

Predictors	Unstandardized Coefficients		Standardized Coefficients β	t	95% CI	*p*
	B	SE				
Constant	0.64	1.86		0.35	[−3.01; 4.30]	0.729
Model of intrapartum care	−0.63	0.34	−0.13	−1.86	[−1.29; 0.04]	0.064
Maternal age	−0.04	0.04	−0.07	−0.96	[−0.11; 0.04]	0.337
Educational level	0.07	0.27	0.02	0.25	[−0.46; 0.59]	0.801
Marital status	0.33	0.34	0.67	0.97	[−0.34; 1.01]	0.334
Parity	0.55	0.34	0.11	1.59	[−0.13; 1.22]	0.112
Number of people providing support	0.27	0.33	0.06	0.81	[−0.39; 0.92]	0.422
Pre-pregnancy body mass index	0.02	0.22	0.01	0.10	[−0.41; 0.45]	0.921

B—unstandardized coefficient; SE—standard error; β—standardized coefficient; CI—confidence interval.

**Table 4:** Changes in breastfeeding self-efficacy between midwife-led and obstetrician-led care groups.

BSES-SF Statement		T1 (Mean ± SD)	T2 (Mean ± SD)	*p* (T1 vs. T2) *	Δ (Mean ± SD)	Cohen’s *d* (Δ)	*p* (Δ Comparison) **
Determine that my baby is getting enough milk	OB-led	3.28 (0.9)	3.72 (1.2)	<0.001	0.44 (1.3)	−0.17	0.013
	MW-led	3.39 (0.9)	4.04 (0.9)	<0.001	0.66 (1.3)		
Successfully cope with breastfeeding like I have with other challenging tasks	OB-led	3.17 (1.0)	3.52 (1.2)	0.011	0.35 (1.5)	−0.16	0.023
	MW-led	3.26 (0.9)	3.87 (1.0)	<0.001	0.57 (1.1)		
Breastfeed my baby without using formula as a supplement	OB-led	3.76 (1.3)	3.94 (1.6)	0.038	0.22 (1.5)	−0.10	0.032
	MW-led	3.46 (1.3)	4.00 (1.4)	0.001	0.40 (1.4)		
Ensure that my baby is properly latched on for the whole feeding	OB-led	3.34 (1.1)	3.45 (1.4)	0.020	0.16 (1.3)	−0.30	0.001
	MW-led	3.46 (1.0)	4.03 (1.1)	<0.001	0.62 (1.2)		
Manage the breastfeeding situation to my satisfaction	OB-led	3.42 (1.0)	3.58 (1.5)	0.029	0.16 (1.2)	−0.25	0.003
	MW-led	3.51 (0.9)	4.09 (1.1)	<0.001	0.51 (1.1)		
Manage to breastfeed even if my baby is crying	OB-led	3.13 (1.0)	3.48 (1.4)	0.002	0.38 (1.4)	−0.23	0.007
	MW-led	3.18 (1.0)	3.85 (1.1)	0.007	0.73 (1.2)		
Keep wanting to breastfeed	OB-led	3.95 (1.0)	3.77 (1.4)	0.037	−0.09 (1.1)	0.01	0.052
	MW-led	4.00 (0.9)	3.98 (1.2)	0.031	−0.11 (1.2)		
Comfortably breastfeed with my family members present	OB-led	3.89 (1.0)	3.51 (1.4)	0.004	−0.26 (1.2)	−0.12	0.021
	MW-led	3.95 (1.0)	3.86 (1.2)	0.007	−0.09 (1.2)		
Be satisfied with my breastfeeding experience	OB-led	3.34 (1.0)	3.59 (1.5)	0.028	0.31 (1.5)	−0.01	0.049
	MW-led	3.46 (1.0)	3.91 (1.1)	0.026	0.32 (1.2)		
Deal with the fact that breastfeeding can be time-consuming	OB-led	3.92 (1.0)	3.77 (1.4)	0.981	−0.01 (1.0)	−0.11	0.034
	MW-led	3.92 (0.9)	4.21 (1.2)	0.001	0.14 (1.2)		
Finish feeding my baby on one breast before switching to the other breast	OB-led	3.74 (0.9)	3.56 (1.5)	0.036	−0.25 (1.3)	−0.48	<0.001
	MW-led	3.69 (1.0)	4.16 (1.1)	<0.001	0.39 (1.2)		
Continue to breastfeed my baby for every feeding	OB-led	3.78 (1.1)	3.92 (1.6)	0.012	0.20 (1.5)	−0.20	0.026
	MW-led	3.79 (0.9)	4.32 (1.1)	0.033	0.50 (1.2)		
Manage to keep up with my baby’s breastfeeding demands	OB-led	3.81 (1.0)	3.88 (1.6)	0.028	0.13 (1.6)	−0.22	0.028
	MW-led	3.69 (1.0)	4.19 (1.1)	0.060	0.46 (1.2)		
Tell when my baby is finished breastfeeding	OB-led	3.62 (1.0)	3.73 (1.4)	0.012	0.22 (1.1)	−0.19	0.022
	MW-led	3.59 (1.0)	4.10 (1.2)	0.007	0.51 (1.3)		
Total	OB-led	50.17 (10.4)	51.42 (18.1)	0.004	1.97 (18.3)	−0.19	0.017
	MW-led	50.38 (9.9)	56.02 (14.1)	<0.001	5.02 (13.6)		

* Wilcoxon signed-rank test; ** Mann–Whitney U test. Unadjusted *p*-values are reported. For item-level comparisons, statistical significance was evaluated using a Bonferroni-adjusted threshold of *p* < 0.00357 (0.05/14). Total BSES-SF score comparisons were evaluated at *p* < 0.05. Paired T1–T2 analyses included 94 women in the MW-led group and 124 women in the OB-led group. T1 and T2 descriptive means are based on all available observations at each time point, whereas Δ scores and paired within-group comparisons are based only on participants with data available at both T1 and T2.

**Table 5:** Multivariable linear regression for changes (Δ T2–T1) in total BSES-SF scores.

Predictors	Unstandardized Coefficients		Standardized Coefficients β	t	95% CI	*p*
	B	SE				
Constant	−12.74	12.21		−1.04	[−36.81; 11.34]	0.298
Model of intrapartum care	4.02	2.21	0.12	1.82	[−0.34; 8.38]	0.071
Maternal age	−0.34	0.25	−0.10	−1.37	[−0.84; 0.15]	0.174
Educational level	−0.47	1.75	−0.02	−0.27	[−3.92; 2.99]	0.790
Marital status	−1.49	2.26	−0.05	−0.66	[−5.94; 2.96]	0.510
Parity	0.81	2.26	0.03	0.36	[−3.65; 5.26]	0.722
Number of people providing support	5.22	2.19	0.16	2.39	[0.91; 9.54]	0.018
Pre-pregnancy body mass index	2.90	1.44	0.14	2.02	[0.07; 5.73]	0.045

B—unstandardized coefficient; SE—standard error; β—standardized coefficient; CI—confidence interval.

**Table 6:** Multivariable linear regression for total BSES-SF scores at T2.

Predictors	Unstandardized Coefficients		Standardized Coefficients β	t	95% CI	*p*
	B	SE				
Constant	44.58	15.52		3.56	[19.90; 69.26]	<0.001
Model of intrapartum care	4.66	2.27	0.14	2.06	[0.19; 9.12]	0.041
Maternal age	−0.44	0.26	−0.12	−1.71	[−0.94; 0.07]	0.089
Educational level	0.47	1.79	0.02	0.26	[−3.05; 3.99]	0.794
Marital status	−2.92	2.32	−0.09	−1.26	[−7.49; 1.64]	0.208
Parity	5.59	2.32	0.17	2.41	[1.03; 10.16]	0.017
Number of people providing support	3.80	2.24	0.11	1.70	[−0.62; 8.22]	0.091
Pre-pregnancy body mass index	1.39	1.47	0.06	0.95	[−1.51; 4.29]	0.346

B—unstandardized coefficient; SE—standard error; β—standardized coefficient; CI—confidence interval.

## Data Availability

The data presented in this study are available on request from the corresponding author due to confidentiality restrictions.

## Abbreviations

The following abbreviations are used in this manuscript:

MW-led	Midwife-led care
OB-led	Obstetrician-led care
PROM	Patient-Reported Outcome Measures
MIBS	Mother–Infant Bonding Scale
BSES-SF	Breastfeeding Self-Efficacy Scale-Short Form
